# The Cause of Poor-Law Reform

**Published:** 1915-07-24

**Authors:** 


### THE CAUSE OF POOR-LAW REFORM.

### Two Typical Needs and the Remedy.

### The Local Government Inquiry at Epping.

We have drawn attention on several occasions to the criticisms which have been directed by the Epping Guardians against Dr. Denning, the medical officer of their workhouse. The dispute led to the Guardians asking for a Local Government Board inquiry into his conduct, and the result of this inquiry has now been published. Mr. Hervey and Dr. Fuller, who conducted the inquiry, report that after careful consideration of the evidence they are of opinion that it has not been shown that there has been any want of care on the part of Dr. Denning in the treatment of his patients, or any neglect of duty or other irregularity on his part, such as to call for the determination of his appointment. They find, however, that Dr. Denning has not fully carried out the duty imposed upon him of making the necessary entries on the patients' record papers, and that he has delegated responsibility to the superintendent nurse in regard to the dispensing of medicines. They regard this latter procedure as open to grave objection, and they expect the requirements of the Poor Law Institutions Order, as regards record-keeping, to be strictly observed in the future. As regards midwifery cases, it appears that attendance has usually been given by the superintendent nurse, although Dr. Denning has given a general supervision to the cases and has been in readiness to attend as and when required. They point out that the medical officer is not entitled to a fee for attendance in such cases unless he has rendered substantial assistance either at or shortly after the birth. Dealing with two specific cases in which the Master accused Dr. Denning of not attending promptly when summoned, the Inspectors hold that, in view of the messages sent, there was no unreasonable delay in the medical officer's attendance. As regards the charge made by Dr. Denning that the Master had failed to provide assistance needed for carrying a patient to the operation room, they regret that this assistance was not made available more promptly.

### The Local Government Board's Decision and the Guardians' Action.

After considering this report the Local Government Board removed Dr. Denning's suspension, and directed that he should forthwith resume the discharge of his duties as medical officer. The Board also expressed the hope that the medical officer and master would in future work harmoniously together. The Inspectors' report and the Local Government Board decision came up for discussion at the last meeting of the Guardians, when a resolution was adopted that twenty-eight days' notice should be given to Dr. Denning to determine his appointment under the board. Previous discussions at the Guardians' meetings have shown that throughout the dispute the majority of them have been strongly prejudiced against Dr. Denning. The resolution they have adopted cannot, of course, be enforced in view of the Local Government Board's decision, and is only another illustration of animus. As we have pointed out before, the whole of the difficulty has arisen from the power of interference in purely medical matters given to the workhouse master by the Poor Law Institutions Order. Until the Order is amended, and the medical officer is given administrative control of the sick wards, friction is bound to arise if the master attempts to exert the authority which the Order gives him over these wards.

### Guardians and Able-Bodied Paupers.

A letter read at the last meeting of the South Dublin Guardians from the Dublin Citizens' Association did not receive the consideration due to it. The Association pointed out that from information it had received the English workhouses had been cleared of able-bodied inmates, who were now making an independent and substantial living. The Association suggested that the Guardians should make inquiries as to the work available, and bring pressure to bear upon all able-bodied inmates to make them earn their living. Individual Guardians pointed out that there were very few able-bodied inmates in their institutions—not enough, indeed, to work their farm—and that most of those who were classed as healthy had some mental or physical defect. The Guardians finally decided to take no action. This, we think, was a grave mistake. At a time like the present there is no reason why even a single able-bodied man should remain in a workhouse, and many defectives who in ordinary times could not compete in the labour market can now find work. It is the duty of every Board of Guardians to investigate carefully the condition and possibilities of every inmate, and to give him or her the opportunity to find work. Where the inmate fails to take advantage of the advice and opportunity offered, stronger measures should be taken. Many of the inmates of Poor-Law institutions are lacking in will-power and initiative. They are capable of working well under direction, but are incapable of seeking for openings for themselves. At the present time Guardians and their officers, if they will only take a little individual trouble in the matter, can place out these inmates in positions where they will become self-supporting, and where they will acquire the habit of self-reliance and the normal love of independence.

